# Tomographic assessment of anatomical boundaries in endoscopic frontal sinusotomy: retrospective radiologic analysis of Draf IIA and IIB approaches

**DOI:** 10.1016/j.bjorl.2026.101787

**Published:** 2026-03-19

**Authors:** Paulo José da Costa Mariz Neto, Camila de Santa Cruz Souza, Matheus Dorigatti Soldatelli, Rainer Guilherme Haetinger, Eduardo Macoto Kosugi, Thiago Freire Pinto Bezerra

**Affiliations:** a Instituto de Medicina Integral Professor Fernando Figueira, Recife, PE, Brazil; b Private Office, Recife, PE, Brazil; c Boston Children’s Hospital, Boston, Massachusetts, United States; d BP Medicina Diagnóstica, Hospital BP ‒ Beneficência Portuguesa de São Paulo, São Paulo, SP, Brazil; e Universidade Federal de São Paulo, Escola Paulista de Medicina, São Paulo, SP, Brazil; f Universidade Federal de Pernambuco, Recife, PE, Brazil

**Keywords:** Frontal sinusotomy, Endoscopic sinus surgery, Sinus anatomy, Chronic rhinosinusitis, Computed tomography

## Abstract

**Objectives:**

The aim of this study was to evaluate the anatomical dimensions and boundaries involved in Draf IIA (DIIA) and Draf IIB (DIIB) frontal sinusotomies, and to determine how complete frontal beak removal affects the frontal sinus drainage pathway. Specifically, we compared the proportional increase in drainage area between DIIA and DIIB performed with and without complete frontal beak removal.

**Methods:**

This retrospective radiological anatomical study analyzed 296 hemifaces (from 148 patients) who underwent paranasal sinus Computed Tomography (CT). Individuals over 18-years of age without evidence of paranasal sinus disease were included. We measured the anatomical distances defining the frontal recess and estimated the differences in frontal recess area for three approaches: Draf type IIA (DIIA), Draf type IIB without complete removal of the frontal beak (DIIBwoFB), and Draf type IIB with complete removal of the frontal beak (DIIB).

**Results:**

The measured areas of the frontal ostium were as follows: 59.00 ± 24.34 mm^2^ (range: 0.55–160.00) for DIIA, 79.66 ± 30.29 mm^2^ (range: 0.85–200.00) for DIIBwoFB, and 147.00 ± 35.25 mm^2^ (range: 71.47–282.74) for DIIB. The proportional increase in drainage area was significantly higher when comparing DIIA to DIIB than to DIIBwoFB (3.64 ± 14.37 [range: 2.52–248.40] vs. 1.37 ± 0.18 [range: 1.00–2.64], respectively).

**Conclusion:**

Among the evaluated surgical approaches, the greatest proportional increase in anatomical drainage area was achieved with Draf IIB combined with complete frontal beak removal. These findings underscore the importance of frontal beak resection when considering the Draf IIB approach.

**Level of evidence:**

IV.

## Highlights

• CT-based measurements guide safer and more effective frontal sinus surgeries.
• First study to objectively measure drainage gain in Draf frontal sinusotomy.
• Incomplete beak removal compromises surgical success in frontal sinusotomy.
• Draf IIB is only effective with full frontal beak removal.
• Complete removal of the frontal beak triples frontal sinus drainage pathway.

## Introduction

The main challenges of endoscopic frontal sinusotomy stem from the difficulty of visualization due to the anterosuperior position of the frontal recess within the nasal cavity. This often necessitates the use of angled endoscopes, advanced surgical techniques, specialized instruments, and precise anatomical knowledge of its boundaries with vital structures such as the orbit, anterior skull base, and anterior ethmoidal artery.^1^, ^2^, ^3^

The frontal recess is considered the most anterosuperior part of the ethmoid sinus, located inferior to the frontal sinus ostium, although this definition remains debated. It is frequently, and inaccurately, used as a synonym for the 'frontal sinus drainage pathway'. In fact, the drainage pathway is more anatomically complex and is influenced by the position of frontoethmoidal cells and the insertion of the uncinate process. The term 'frontal sinus ostium' is also incorrect, as it implies a two-dimensional structure. The term ‘nasofrontal duct’ or ‘frontonasal duct’ is no longer used because the frontal sinus drainage pathway does not function as an actual duct.^2^, ^3^, ^4^, ^5^

The frontal sinus opening is best visualized in the sagittal plane on Computed Tomography (CT), where the contours of the frontal sinus and frontal recess resemble an hourglass shape, with the narrowest part representing the junction between the frontal sinus and the recess. On the other hand, the frontal recess is the drainage space of the frontal sinus and contains a variable number of frontoethmoidal cells. The medial boundary of the frontal recess is formed by the lateral lamella of the cribriform plate and the vertical lamella of the middle turbinate. Its lateral boundary is formed by the lamina papyracea and the lacrimal bone. The posterior boundary is the anterior wall of the ethmoid bulla, and the anterior boundary is the Frontal Beak (FB) and the agger nasi. The FB was defined as the superomedial portion of the frontal bone that forms the anterior boundary of the frontal recess, without inclusion of the frontal process of the maxilla or the nasal bone.^6^, ^7^, ^8^, ^9^

Historically, frontal sinus approaches, including Lynch and Lothrop procedures, have shown a long-term surgical failure rate of 30%. To minimize potential surgical failures, Draf systematized different interventions for frontal sinuses. The Draf classification system categorizes endoscopic frontal sinusotomies into four types based on the extent of anatomical removal: Draf I: limited to anterior ethmoidectomy without direct intervention in the frontal recess. Draf IIa: complete clearance of the frontal recess while preserving the frontal sinus floor and intersinus septum. Draf IIb: removal of the frontal sinus floor and medial wall to allow access into the frontal sinus, typically unilaterally.

Draf III (Modified Lothrop Procedure): extended bilateral frontal sinusotomy through resection of the intersinus septum and frontal sinus floor via a trans-septal approach.

Draf Type II A (DIIA) intervention is indicated for a variety of conditions where the frontal recess approach is sufficient to promote functional drainage of the frontal sinus, such as most cases of chronic frontal sinusitis. It involves a complete approach to the cells and lamellae occupying the frontal recess while respecting their bony boundaries. Thus, the maximum drainage obtained in DIIA corresponds to the natural limit of the frontal recess.

Draf Type II B (DIIB) aims to expand the drainage of the frontal sinus beyond the natural limits of the frontal recess. This requires the removal of part of the bony boundaries, particularly the floor of the sinus, including the FB, which is typically done using drills due to the thickness and resistance of the involved bone. DIIB increases the drainage diameter and allows access for instrumentation within the frontal sinus, which is necessary in cases such as tumors when tumor insertion needs to be addressed. It is also more suitable for non-inflammatory conditions, as drills can induce neo-osteogenesis in inflammatory diseases.^10^^,^^11^

The primary objective of this study was to quantify the anatomical space and boundaries associated with Draf type IIA and Draf type IIB frontal sinus surgery, using high-resolution computed tomography as a static anatomical model. A secondary objective was to determine the proportional change in the frontal sinus drainage pathway area resulting from complete frontal beak removal. We hypothesized that complete removal of the frontal beak in Draf type IIB provides a significantly greater drainage area compared with both Draf type IIA and Draf type IIB without full frontal beak removal, potentially offering relevant anatomical advantages for surgical planning.

## Methods

This research was approved by the Ethics and Research Committee of Beneficência Portuguesa Hospital of São Paulo under protocol number [5.049.943]. All procedures were conducted in accordance with Resolution 466/2012 of the Brazilian Ministry of Health and with the principles of the Declaration of Helsinki, which govern research involving human subjects.

This was a retrospective radiological anatomical study based on Computed Tomography (CT) scans of the paranasal sinuses from asymptomatic adult individuals. Scans were retrieved from the radiology database of Beneficência Portuguesa Hospital of São Paulo, covering the period between August and September 2021. All images were acquired for reasons unrelated to sinonasal disease and showed no radiological evidence of sinonasal pathology.

The inclusion criteria were adults over 18-years of age with no frontal sinus disease, and the exclusion criteria were patients with previous paranasal surgery, trauma, facial deformities, malignant paranasal disease, or congenital malformations.

The study was designed to estimate potential anatomical drainage area gains associated with different frontal sinusotomy approaches. All measurements were theoretical, based on normal anatomy, and do not represent actual postoperative results or healing outcomes. This study was not clinical, interventional, or a descriptive cross-sectional design.

The CT images were obtained using multi-detector tomographs: Siemens Somatom Force, Somatom Definition plus, Somatom Perspective, as well as General Electric VCT, with 0.6 mm slice thickness and multiplanar reconstructions. Post-processing was performed using Carestream software.

**Fig. 1:**
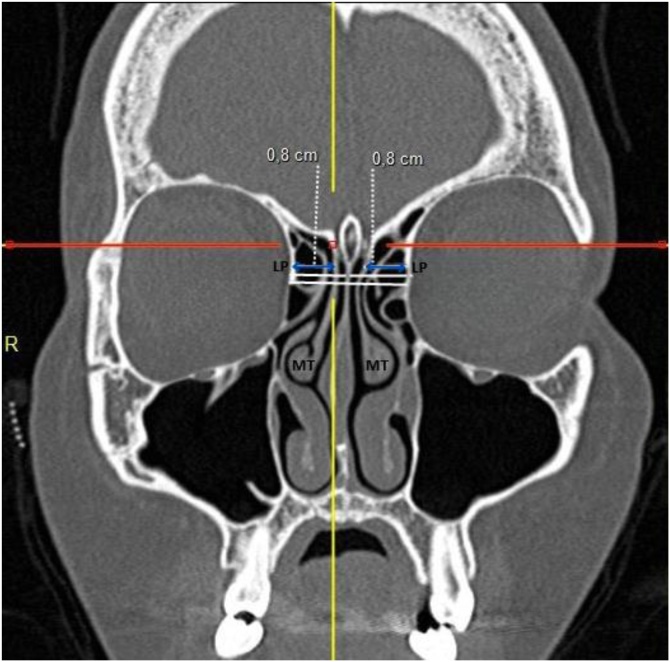
Coronal CT view showing the distance between the Lamina Papyracea (LP) and the vertical portion of the Middle Turbinate (MT), representing the lateral boundary of the frontal recess for Draf type IIA procedures.

The images of eligible patients were evaluated by the researchers, and measurements of anatomical spaces and boundaries for DIIA and DIIB frontal sinusotomies were taken:

I ‒ Anteroposterior distances: a) Distance between the most anterior portion of the cribriform plate and the outer limit of the frontal bone; b) Thickness between the skin and the posterior limit of the FB in the same sagittal plane as measurement (a) (Fig. 1).

II ‒ Lateral distances: (a) Distance between the lamina papyracea at the level of the most anterior portion of the cribriform plate and the medial surface of the vertical portion of the middle turbinate; (b) Distance between the lamina papyracea and the nasal septum (Fig. 1).

III ‒ Based on these measurements, we calculated a safety area for DIIA and DIIB frontal sinusotomies using the formula for calculating the area of an ellipse: anteroposterior distance/2 (sagittal plane) × lateral distance/2 (transverse plane) × π(3.1416). These measurement points were selected to reflect the key anatomical boundaries involved in DIIA and IIB procedures, particularly the lateral and anteroposterior limits of the frontal recess. The lamina papyracea, middle turbinate, cribriform plate, and frontal beak were chosen due to their direct relevance during surgical dissection and consistency with prior anatomical and radiologic studies on frontal sinus surgery. These regions define the extent of bone removal required in each Draf type and are critical for understanding the potential anatomical gain achieved with each technique.

IV ‒ The estimation of the proportional increase in drainage area was performed to compare DIIA and DIIB frontal sinusotomies. Since all measurements were obtained using computed tomography in a standardized way, and height and time were not variables in this analysis, we assumed that the ratio of drainage areas between approaches reflects the potential anatomical gain provided by each technique. This was expressed as a ratio between the calculated drainage areas of DIIB and DIIA, as shown in Table 1.

**Table 1:** Anatomical drainage area ratio.

Flow (Q) = Volume/time = Area × height / time
$\text{R}\text{a}\text{t}\text{i}\text{o}\text{o}\text{f}\text{f}\text{l}\text{o}\text{w}\text{s}=\frac{\text{A}\text{r}\text{e}\text{a}\text{o}\text{f}\text{D}\text{r}\text{a}\text{f}\text{t}\text{y}\text{p}\text{e}\text{I}\text{I}\text{B}\times \text{h}\text{e}\text{i}\text{g}\text{h}\text{t}/\text{t}\text{i}\text{m}\text{e}}{\text{A}\text{r}\text{e}\text{a}\text{o}\text{f}\text{D}\text{r}\text{a}\text{f}\text{t}\text{y}\text{p}\text{e}\text{I}\text{I}\text{A}\times \text{h}\text{e}\text{i}\text{g}\text{h}\text{t}/\text{t}\text{i}\text{m}\text{e}}$=$\frac{\text{A}\text{r}\text{e}\text{a}\text{o}\text{f}\text{D}\text{r}\text{a}\text{f}\text{t}\text{y}\text{p}\text{e}\text{I}\text{I}\text{B}}{\text{A}\text{r}\text{e}\text{a}\text{o}\text{f}\text{D}\text{r}\text{a}\text{f}\text{t}\text{y}\text{p}\text{e}\text{I}\text{I}\text{A}}$

The values were measured five times for each structure, and the highest and lowest values were discarded. All statistical analyses were performed using SAS OnDemand Studio (SAS Institute Inc., Cary, NC, USA). The normality of data distribution was assessed using the Kolmogorov–Smirnov test. For each measurement, the mean, standard deviation, minimum, and maximum values were calculated.

Comparisons between groups were made using the Student’s *t*-test for independent samples, as the aim was to evaluate differences between pairs of groups (e.g., DIIA vs. DIIBwoFB, DIIA vs. DIIB). A significance level of p < 0.05 was adopted.

**Fig. 2:**
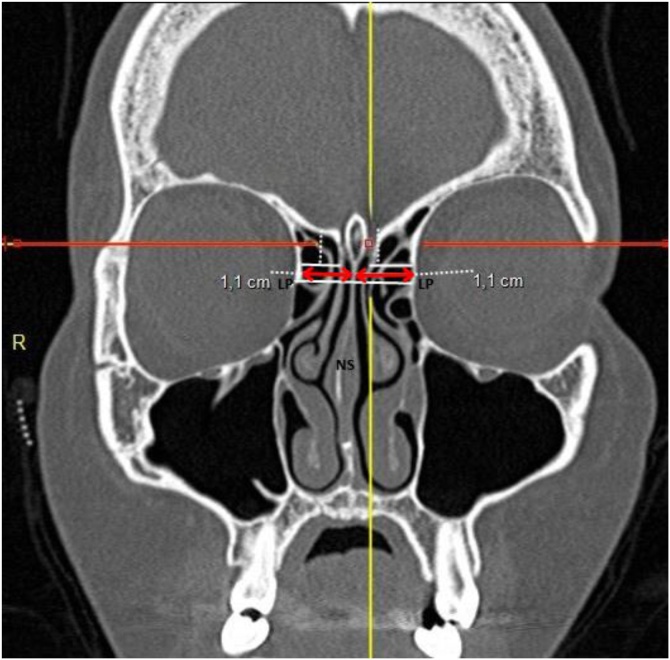
Coronal CT view showing the distance between the Lamina Papyracea (LP) and the Nasal Septum (NS), representing the maximum lateral expansion considered in Draf type IIB approaches.

**Fig. 3:**
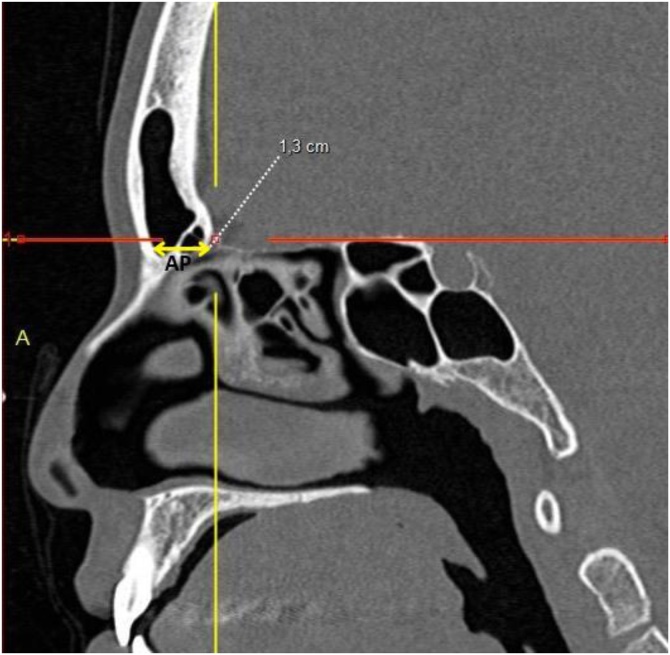
Sagittal view illustrating the Anteroposterior distance (AP) between the anterior limit of the cribriform and the outer border of the frontal bone, defining the depth of the frontal recess.

**Fig. 4:**
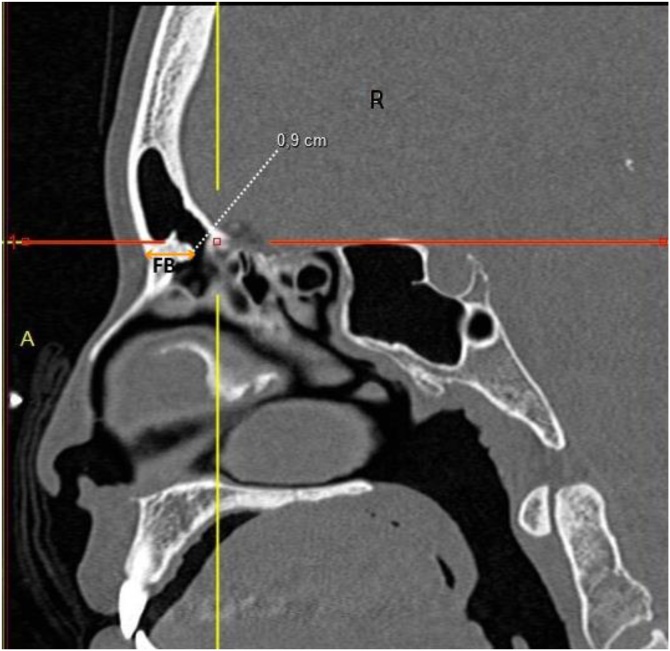
Sagittal CT view illustrating the thickness of the Frontal Beak (FB), which is subtracted from the anteroposterior distance to calculate the area available for drainage in Draf type IIB.

Computed tomography of the facial sinuses in coronal (Fig. 1, Fig. 2) and sagittal (Fig. 3, Fig. 4) views, using a bone window.

## Results

We evaluated 296 hemifaces (148 patients), with a male-to-female ratio of 0.90 (140/156), and a mean age of 45.45 ± 16.87 years (range: 18–87-years).

The lateral distance between the lamina papyracea and the middle turbinate in the coronal view was 8.4 ± 1.5 mm (range: 4.0–14.1 mm), while the distance between the lamina papyracea and the nasal septum was 11.3 ± 1.5 mm (range: 7.0–16.0 mm) (Table 2).

**Table 2:** Estimated cross-sectional areas obtained through simulated Draf type IIA and IIB frontal sinusotomy approaches, with and without complete removal of the frontal beak. Values are expressed as mean ± SD (range).

Measurement	Mean	Standard Deviation	Min	Max
Distance between lamina papyracea and middle turbinate (cm)	0.839	0.156	0.400	1.410
Distance between lamina papyracea and nasal septum (cm)	1.129	0.147	0.700	1.600
Anteroposterior distance (cm)	1.653	0.281	1.100	2.700
Anteroposterior distance minus FB thickness (cm)	0.890	0.288	0.010	2.000
Draf II A area (cm^2^)	0.592	0.243	0.006	1.602
Draf II B area with “Frontal Beak” removal (cm^2^)	1.474	0.352	0.715	2.827
Draf II B area without “Frontal Beak” removal (cm^2^)	0.797	0.303	0.008	2.003
Proportional increase in drainage area between Draf IIA and Draf IIB	3.637	14.365	1.519	248.400
Proportional increase in drainage area between Draf IIA and Draf IIB without frontal beak removal	1.369	0.183	1.000	2.644

Statistical comparisons between groups were performed using Student’s *t*-test for independent samples.

Significant differences were found between approaches (p < 0.01).

The anteroposterior distance between the most anterior portion of the cribriform plate and the outer limit of the frontal bone, measured in the sagittal plane, was 16.5 ± 2.8 mm (range: 11.0–27.0 mm), whereas the anteroposterior distance minus the thickness of the frontal beak was 8.9 ± 2.9 mm (range: 0.1–20.0 mm).

The drainage area for DIIA was 59 ± 24 mm^2^ (range: 1–160 mm^2^), for DIIBwoFB was 79 ± 30 mm^2^ (range: 1–200 mm^2^), and for DIIB was 147 ± 35 mm^2^ (range: 71–282 mm^2^).

The drainage area ratio between DIIB with complete frontal beak removal and DIIA was statistically and clinically significantly higher than that between DIIB without complete removal and DIIA (3.64 ± 14.37 [1.52–248.40] vs. 1.37 ± 0.18 [1.00–2.64], p < 0.01).

## Discussion

This study demonstrates the critical importance of complete frontal beak removal, evidenced by a statistically significant increase in the proportional drainage area when comparing Draf type IIB (DIIB) to Draf type IIA (DIIA) (DIIB/DIIA: 3.64 ± 14.37 [1.52–248.40] vs. DIIB without frontal beak removal/DIIA: 1.37 ± 0.18 [1.00–2.64], p < 0.01). To our knowledge, no previous publications have directly compared the anatomical gain in drainage area between DIIA and DIIB with and without complete frontal beak removal using radiological simulation. Our results indicate that DIIB confers a clinically relevant increase in the drainage pathway only when the frontal beak is adequately resected.

The gains observed in this investigation represent anatomical space increases measured via Computed Tomography (CT) and should not be interpreted as direct measures of airflow or mucociliary drainage. This model offers a theoretical estimation of the drainage pathway potentially available after different surgical approaches, without incorporating postoperative variables such as scarring, edema, or inflammation. Accordingly, these values are intended to guide preoperative planning rather than to predict surgical outcomes.

Our findings further reveal a marked difference in area between DIIA (59.0 ± 24.0 mm^2^) and DIIB (147.0 ± 35.0 mm^2^). DIIB inherently requires frontal beak removal, typically performed using a drill. In our simulation, preservation of the frontal beak reduced the frontal recess area from 147.35 ± 35.25 mm^2^ to 79.66 ± 30.29 mm^2^. These results reinforce the anatomical and surgical principles described by Draf, who emphasized that successful frontal sinusotomy (types II and III) depends on wide exposure and sufficient bone removal to maintain long-term patency and ventilation.^16^

Thus, the justification for a DIIB approach extends beyond increasing the lateral-medial dimension from 0.84 ± 0.15 cm (0.40–1.41) to 1.13 ± 0.15 cm (0.70–1.60); it also requires adequate anteroposterior enlargement from 59.0 ± 24.0 mm^2^ (10–160 mm^2^) to 147.0 ± 35.0 mm^2^ (71–283 mm^2^) through complete frontal beak removal.

The lateral-medial dimension of the frontal recess, measured as the distance between the lamina papyracea and the middle turbinate, averaged 0.84 ± 0.16 cm (0.40–1.41), while the anteroposterior distance (excluding frontal beak thickness) averaged 0.896 ± 0.28 cm. Chandra et al.^14^ suggested that an opening of approximately 4–5 mm is sufficient to achieve a patent frontal sinus, a value substantially lower than the 1.65 ± 0.28 cm observed in our cohort. Naidoo et al.^12^ reported that smaller lateral-medial and anteroposterior distances in DIIA procedures were associated with postoperative stenosis, while Korban and Casiano^13^ recommended an anteroposterior distance of 6–8 mm for improved outcomes. Dassi et al.^15^ underscored the complex variability of frontal sinus anatomy, advocating that preoperative imaging guide the surgical extent.

These prior studies^12^, ^13^, ^14^, ^15^, ^16^ differ from ours in methodology: Naidoo, Korban and Casiano, and Chandra used an indirect postoperative measurement technique, introducing a 4 mm instrument endoscopically to assess patency. While this approach permits direct visualization of edema, scarring, and polyps, its accuracy is limited by the small size of the frontal recess, and measurement discrepancies can markedly affect results. Conversely, our CT-based dimensions suggest that a complete lateral-medial opening of the magnitude observed herein may reduce the risk of postoperative stenosis.

Becker et al.^17^ reported a mean frontal sinus opening area of 47.5 ± 21.1 mm^2^ for DIIA and 105.1 ± 45.6 mm^2^ for DIIB using intraoperative endoscopy ‒ both smaller than our CT-based findings of 59.23 ± 24.34 mm^2^ and 147.35 ± 35.25 mm^2^, respectively. Such discrepancies likely reflect methodological limitations of intraoperative endoscopic measurements compared to radiological simulations. While CT-based modeling may overestimate the actual intraoperative size, it also highlights the potential for safely expanding the frontal sinus opening.

Importantly, a larger opening does not inherently guarantee better clinical outcomes. Dhepnorrarat et al.^18^ observed restenosis rates of 23% in DIIB vs. 3.6% in DIIA for frontoethmoidal mucoceles, though without statistical significance; this may be attributable to selection bias, as DIIB was likely chosen for anatomically predisposed cases. Furthermore, the removal of the frontal beak entails more extensive bone work, which may induce neo-osteogenesis and thereby increase stenosis risk. For these reasons, Draf type III may be preferred when DIIA proves inadequate, as the decision to perform DIIB should not rest solely on anatomical metrics.

It is also plausible that the underlying inflammatory status of the mucosa significantly influences surgical success or failure. Naidoo et al.^12^ demonstrated similar frontal sinusotomy patency rates regardless of rhinosinusitis etiology ‒ 100% for mucoceles and 88.4% for chronic rhinosinusitis with polyps ‒ suggesting that sinusotomy size may exert a greater impact on outcome than mucosal disease severity.

Preoperative CT remains indispensable for surgical planning in light of the frontal sinus’s complex anatomy. To our knowledge, no previous studies have reported preoperative tomographic distance measurements directly comparable to our data. Such measurements can delineate the critical anatomical boundaries for frontal recess dissection, potentially enhancing surgical precision. In agreement with Dassi et al.,^15^ our findings support the use of CT-based anatomical benchmarks to guide surgical extent and mitigate complications.

A limitation of our work lies in its reliance on CT as a static anatomical model, which cannot account for postoperative changes such as mucosal healing, bony remodeling, or inflammation. As a radiological modeling analysis based on asymptomatic individuals without intraoperative correlation, our findings should be interpreted as theoretical simulations intended to inform surgical planning rather than predict clinical outcomes. Future investigations integrating pre- and postoperative CT data, coupled with long-term follow-up of patients undergoing Draf procedures, will be essential to determine whether the anatomical gains observed here translate into improved patency and surgical success.

## Conclusion

Complete removal of the frontal beak results in a substantially greater proportional increase in the anatomical drainage area of the frontal sinus, from 1.37 ± 0.18 in DIIA to 3.64 ± 14.37 in Draf type IIB. Therefore, the decision to perform this procedure should always include preoperative planning for complete frontal beak resection. In many cases, only after proper execution of this surgical step should the procedure be considered unsuccessful, at which point more extensive approaches, such as Draf type III, may be warranted.

## ORCID ID

Camila de Santa Cruz Souza: 0000-0001-5123-9626

Rainer Guilherme Haetinger: 0000-0001-9914-2030

Thiago Freire Pinto Bezerra: 0000-0003-3420-5074

## Funding

The authors declare that this research did not receive any specific grant from funding agencies in the public, commercial, or not-for-profit sectors.

## Data availability statement

The authors declare that all data are available in repository.

## Declaration of competing interest

The authors declare no conflicts of interest.
